# Case of Rupture of the Diaphragm in a Horse

**Published:** 1842-12

**Authors:** 


*Case of Rupture of the Diaphragm in a Horse.*—On the 8th of January last, Mr. Cope, whose pupil I have the honor to be, was desired to look at a black mare, the property of a farmer in this county, who said that, in coming up one of the steep hills that are so plentiful in the county, she stopped suddenly and breathed very laboriously, so that he could hear her at a great distance off. She was unable to proceed for full fifteen minutes, and then she slowly journeyed on with her load, which was upwards of a ton. On coming under our care the following symptoms were presented:—

She was constantly lying down and getting up again; while down she would frequently turn her head to her side; she perspired very much; the pulse was 80, and hard; the extremities moderately warm.

Venesection was had recourse to immediately, and copiously; and opium, combined with ol. lini, was administered; but in spite of our efforts she died in about six hours after the first appearance of illness.

It would be useless to occupy much time in describing the post-mortem appearances; suffice it to say, that on examining the chest the diaphragm was found to be ruptured. The rupture extended about seven inches, and a large portion of the intestines had protruded through the opening. They were very considerably inflamed, and this rupture was doubtless the cause of death.—*Mr. Osborne, in Veterinarian.*

